# Population Genetic Analysis of *Paris polyphylla* var. *yunnanensis* Based on cpDNA Fragments

**DOI:** 10.3390/genes14091754

**Published:** 2023-09-02

**Authors:** Dan Wang, Yu Huang, Lu Rui, Huihui Du, Junsheng Qi, Mingguo Ma, Nong Zhou

**Affiliations:** 1 The Chongqing Engineering Laboratory for Green Cultivation and Deep Processing of Three Gorges Reservoir Area’s Medicinal Herbs, College of Food and Biology Engineering, Chongqing Three Gorges University, Chongqing 404120, China; wd21171103@163.com (D.W.); information93@163.com (Y.H.); 20200016@sanxiau.edu.cn (L.R.); duhuihui2010@163.com (H.D.); qijunsheng@163.com (J.Q.); 2 College of Pharmacy, Dali University, Dali 671000, China; 3 Research Center of Biomass Clean Utilization, Beijing Key Laboratory of Lignocellulosic Chemistry, College of Materials Science and Technology, Beijing Forestry University, Beijing 100083, China

**Keywords:** *Paris polyphylla* var. *yunnanensis*, cpDNA fragment, genetic diversity, genetic distance

## Abstract

*Paris polyphylla* var. *yunnanensis* is a well-known medicinal plant that is mainly distributed in Southwest China; however, its genetic diversity and biodiversity processes are poorly understood. In this study, the sequences of cpDNA *trn*L-*trn*F fragments of 15 wild populations and 17 cultivated populations of *P. polyphylla* var. *yunnanensis* were amplified, sequenced, and aligned to study the population genetics of this species. Genetic diversity was analyzed based on nucleotide diversity, haplotype diversity, Watterson diversity, population-level diversity, and species-level genetic diversity. Genetic structure and genetic differentiation were explored using haplotype distribution maps and genetic distance matrices. A total of 15 haplotypes were identified in the 32 populations of *P. polyphylla* var. *yunnanensis*. Five unique haplotypes were identified from the fourteen haplotypes of the cultivated populations, while only one unique haplotype was identified from the ten haplotypes of the wild populations. The haplotype richness and genetic diversity of the cultivated populations were higher than those of the wild populations (*H_T_* = 0.900 vs. 0.861). In addition, there were no statistically significant correlations between geographic distance and genetic distance in the cultivated populations (r = 0.16, *p* > 0.05), whereas there was a significant correlation between geographical distance and genetic structure in the wild populations (r = 0.32, *p* > 0.05), indicating that there was a geographical and genetic connection between the wild populations. There was only 2.5% genetic variation between the wild populations and cultivated populations, indicating no obvious genetic differentiation between the wild and cultivated populations. Overall, the genetic background of the cultivated populations was complex, and it was hypothesized that the unique haplotypes and higher diversity of the cultivated populations were caused by the mixed provenance of the cultivated populations.

## 1. Introduction

*P. polyphylla* var. *yunnanensis* is a perennial herb whose stem is generally 20–30 cm in length and purplish red in color, and the plant has a membranous leaf sheath at the base that embraces the stem. The pedicel protrudes from the top of the stem and bears a terminal flower. The flowering period is from June to July, and the fruiting period is from September to October. This plant is mainly found in Southwest China (provinces of Sichuan, Guizhou, Yunnan, and Guangxi); prefers acidic or slightly acidic soil with rich humus layers; and is suitable for growing in evergreen broad-leaved forests, bamboo forests, or shrublands with temperatures in the range of 16–28 °C and elevations ranging from 1400 m to 3100 m [1]. Pharmacological studies of *P. polyphylla* var. *yunnanensis* have shown that the main functional components of this plant are Paris saponin and diosgenin, which have demonstrated antitumor, antibacterial, antiviral, and hemostatic functions [2,3]. *P. polyphylla* var. *yunnanensis* has high medicinal and economic value and is the natural raw material that comprises 81 different Chinese patent medicines, such as “Yunnan Baiyao” and “Gongxuening Capsule” [4], corroborating its role in the development of traditional Chinese medicines (TCMs) for tumor treatment, immunomodulation, and others [5]. *P. polyphylla* var. *yunnanensis* is listed in the *Pharmacopoeia of the People’s Republic of China* as one of the source plants of *Paris polyphylla*. However, wild *P. polyphylla* var. *yunnanensis* suffers from slow growth, and it has been overexploited, making it difficult to obtain sufficient numbers of seedlings. Many farms collect wild *P. polyphylla* var. *yunnanensis* and cultivate its samples to meet the growing market demand. Despite its popularity, few studies have analyzed the genetic diversity of wild and cultivated *P. polyphylla* var. *yunnanensis,* which is important to its future survival. As a consequence, it is urgent to understand the genetic diversity and genetic structure of *P. polyphylla* var. *yunnanensis*. 

The harsh and unstable climate of the Quaternary Ice Age caused large-scale migration of organisms that led to disastrous survival rates. After the Ice Age, the glacial refugium provided a refuge for organisms to survive and ultimately rediffuse to other geographical regions because of its unique geographical conditions. *P. polyphylla* var. *yunnanensis* also underwent this migration, ultimately surviving and differentiating well in Southwest China. As a result, the genetic diversity and structure of *P. polyphylla* var. *yunnanensis* was deeply affected by the unique geographical conditions of Southwest China. Some studies have sought to identify the molecular markers that elucidate the genetic diversity of *P. polyphylla* var. *yunnanensis*. A total of 153 individuals from six populations (three wild populations and three cultivated populations) in the Sichuan and Yunnan provinces (China) were investigated based on ISSR molecular markers. The genetic diversity of the cultivated populations was higher than that of the wild populations (0.153 vs. 0.151), and there was no obvious genetic differentiation between the cultivated populations and the wild populations [6]. The genetic diversity of 62 individuals from the Yunnan province was explored based on SSR molecular markers. Ten pairs of primers were used as single-sequence-repeat (SSR) markers, and a heterozygosity range of 0.790–0.976 was observed following the analysis [7]. SSR molecular markers were also utilized to analyze the genetic diversity of 115 samples from five populations in Yunnan province, and the as-expected heterozygosity was 0.7744 at the species level and 0.6548 at the population level, indicating a high genetic diversity level of *P. polyphylla* var. *Yunnanensis* [8]. Using amplified fragment length polymorphism (AFLP) molecular markers, the genetic diversity at the species level and population level was 0.2768 and 0.1821, respectively, indicating that the 15 populations of *P. polyphylla* var. *Yunnanensis* in the Yunnan province had a low genetic diversity [9]. AFLPs were used in the genetic analysis of 15 wild and 17 cultivated populations of *P. polyphylla* var. *yunnanensis* [10], which revealed that the cultivated populations had higher genetic diversity than the wild populations at the species level (HE = 0.2636 vs. 0.2616, respectively). 

Chloroplast genome (cpDNA) is maternally inherited in most angiosperms, while it is paternally inherited in most gymnosperms, meaning it undergoes almost no recombination. Compared to nuclear genomic markers inherited from both parents, cpDNA markers have the ability to reveal genetic differences in small population sizes. Therefore, cpDNA markers serve as an effective tool for studying phylogeography and elucidating the migration and diffusion routes of species in shelters and post-glacial periods [11,12]. Although some population genetic studies of *P. polyphylla* var. *yunnanensis* had been reported, earlier studies that focused on the Yunnan province had a narrow research area and only studied a small number of populations, which did not effectively reflect the genetic diversity and genetic structure of *P. polyphylla* var. *yunnanensis* at the entire species level. In addition, information regarding the phylogenetic structure of *P. polyphylla* var. *yunnanensis* based on cpDNA and nuclear genes has not yet been reported. 

The purpose of the present study was to analyze the genetic diversity and structure of 15 wild populations and 17 cultivated populations of *P. polyphylla* var. *yunnanensis* using cpDNA fragments (*trn*L–*trn*F).

## 2. Materials and Methods

### 2.1. Materials and Reagents

In the *trn*L–*trn*F experiment using cpDNA fragments, the as-used materials were the same as AFLP markers [10]. There were 364 effective individuals from 32 populations, including 15 wild and 17 cultivated populations. 

### 2.2. PCR Amplification and Sequencing of cpDNA trnL–trnF Fragments

#### 2.2.1. Total DNA Extraction and Purification 

The DNA from approximately 0.5 g of dried *P. polyphylla* var. *yunnanensis* leaves from each sample was extracted using cetyltrimethylammonium bromide (CTAB) according to a previously published (and improved) method [13]. 

#### 2.2.2. PCR Amplification and Sequencing of cpDNA *trn*L-*trn*F Fragments 

PCR amplification and sequencing were performed on 364 individuals using screened primers of cpDNA *trn*L-*trn*F (base sequence of C: 5′-CGAAATCGGTAGACGCTACG-3′, F: 5′-ATTTGAACTGGTGACACGAG-3′). PCR amplification of the *trn*L-*trn*F fragments was performed in a 20 µL reaction system using a K960 PCR apparatus with the following thermocycling program: 95 °C for 5 min, 95 °C for 1 min, 58 °C for 1 min, 72 °C for 1 min, and 33 cycles at 72 °C for 10 min; afterward, the PCR products were stored at 4 °C. The PCR products were then electrophoresed over a 1% agarose gel, and the clear bands with no impurities were selected and sent to Beijing Boyoushun Biotechnology Co., Ltd. (Beijing, China) for sequencing.

### 2.3. DNA Sequence Splicing and Comparison

SeqMan 17.1(Lasergene Genomics software package, DNASTAR) was used to check the peak map of all individual sequenced sequences. The positive and negative primers were spliced together and saved in the “.seq” format. Then, the chloroplast haplotypes were determined based on the variations in the sequences; the haplotypes in the population were named “H1”, “H2”, “H3”.... Then, all haplotype sequences saved in the “.seq” format were imported into SeqMan again and saved as a FASTA file for comparison and sequencing (the FASTA files of the sequences of all individuals in the wild populations and cultivated populations were formatted the same way). Finally, the FASTA files were imported into the software for comparison, and the matched sequences were saved as FASTA and NEXUS files for subsequent sequence alignment analysis using MEGA-X [14].

### 2.4. Genetic Diversity Analysis

Genetic diversity parameters based on the DNA sequences included nucleotide diversity (*π*), haplotype diversity (*H_d_*), Watterson diversity (*θ*), population-level genetic diversity (*H_S_*), and species-level genetic diversity (*H_T_*) [15,16]. To determine *π*, two sequences were randomly selected from the samples, and the average nucleotide variation was calculated based on the average unit site. *H_d_* referred to the frequency of two different haplotype sequences randomly selected from the samples. *H_S_* and *H_T_* referred to the haplotype diversity at the population level and species level, respectively. The parameters *π* and *H_d_* were statistically calculated using analysis of molecular variance (AMOVA) tests, while *H_S_* and *H_T_* were calculated using Permut v.1.2.1 with 1000 permutations [17,18]. The genetic diversities of the wild populations, cultivated populations, and all populations together were calculated.

### 2.5. Genetic Structure and Genetic Differentiation Analysis

The haplotype distribution maps of the wild and cultivated populations were edited using the ArcGIS software (v. 10.5) to visualize the distribution of the haplotypes in the wild and cultivated populations. The genetic distances between the wild and cultivated populations were calculated using MEGA-X based on Kimura’s 2-parameter model, which sorted out the geographic distance and genetic distance matrix, and the data were analyzed using GenAlEx (v. 6.503) to perform Mantel tests [19,20]. The AMOVA test was conducted using Arlequin (v. 3.5) to analyze the genetic differentiation of the wild and cultivated populations [18], with 1000 substitutions being used for the significance test. The spatial genetic structure of all populations, including the wild and cultivated populations, was analyzed using the SAMOVA 1.0 (Spatial Analysis of Molecular Variance) software. The software clustered the populations, which were similar in geography and genetics, into groups according to the haplotype composition and geographical distribution of the populations. The K values were simulated from 2 to 10 and annealed 100 times. Each K value had a corresponding intergroup genetic differentiation value (*F_CT_*); when the *F_CT_* value reached a maximum or stabilized at a specific value without a single population, the corresponding K value was considered the best group.

The *G_ST_* and *N_ST_* of the wild and cultivated populations were calculated using Permut v. 1.2.1 (1000 permutations). *G_ST_* is the genetic differentiation coefficient of the frequency of haplotypes (range of 0–1). The smaller the coefficient, the lower the degree of genetic differentiation between populations, indicating that genetic variation mainly exists within each population. *N_ST_* not only considers the frequency of the haplotypes but also the genetic differentiation coefficient related to the genetic distance between haplotypes. In many cases, these two values are not equal. One of the reasons for the difference may be the genetic and geographical structure of the populations. Therefore, the magnitude of the difference could be used to judge the genetic and geographical relationship between populations. When the value of *N_ST_* is significantly greater than the value of *G_ST_*, there is a clear differentiation in the geographical genetic structure between populations. However, when the value of *N_ST_* is equal to or less than the value of *G_ST_*, there is no geographical relationship and no differentiation in genetic structure between populations.

## 3. Results

### 3.1. Genetic Diversity Analysis of P. polyphylla var. yunnanensis

In this study, the cpDNA *trn*L–*trn*F intergenic spacer was used to classify 15 wild populations and 17 cultivated populations of *P. polyphylla* var. *yunnanensis*. Based on the sequence alignment, 15 haplotypes were detected in these 32 populations of *P. polyphylla* var. *yunnanensis.* Five unique haplotypes were identified from the fourteen haplotypes of the cultivated populations, while only one unique haplotype was identified from the ten haplotypes of the wild populations (Table S1). The wild populations of *P. polyphylla* var. *yunnanensis* displayed high genetic diversity at the species level (*H_T_* = 0.861) (Table 1), while the genetic diversity at the population level was low (*H_S_* = 0.135), resulting in a high genetic differentiation among populations (*N_ST_* = 0.919 and *G_ST_* = 0.843). The cultivated populations showed the same trend as the wild populations of *P. polyphylla* var. *yunnanensis* (*H _T_* = 0.900, *H_S_* = 0.222, *N_ST_* = 0.815, and *G_ST_* = 0.754). All populations of *P. polyphylla* var. *yunnanensis* displayed high genetic diversity at the species level (*H_T_* = 0.866), while the genetic diversity at the population level was low (*H_S_* = 0.181), resulting in a high genetic differentiation among populations (*G_ST_* = 0.791 and *N_ST_* = 0.860). Therefore, the total genetic diversity, haplotype diversity, and nucleotide diversity results indicated that the genetic diversity of the wild populations was slightly lower than the diversity of the cultivated populations, suggesting that artificial domestication in different planting regions increased the diversity of *P. polyphylla* var. *yunnanensis*.

### 3.2. Genetic Structure and Genetic Differentiation Analysis of P. polyphylla var. yunnanensis

**Figure 1:**
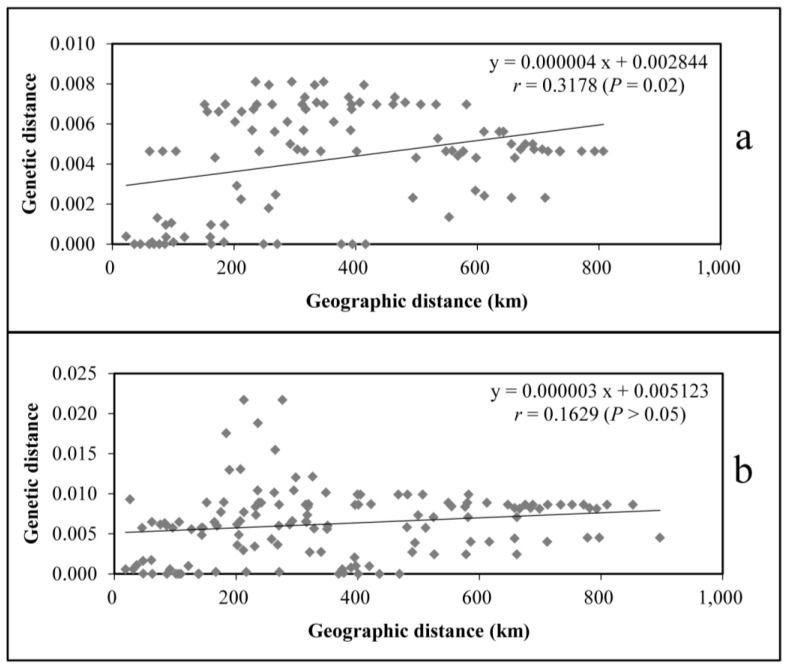
Mantel test analysis: (**a**) Mantel test of wild populations and (**b**) Mantel test of cultivated populations.

In a previous study, the haplotypes determined based on the genetic sequences of the *trn*L–*trn*F fragments were used to generate geographical distribution maps of the haplotypes of wild populations [21]. The Guizhou, Central Yunnan, and Western Yunnan provinces had their own haplotypes without crossing. There was a geographical genetic structure among the wild populations. Additionally, the results of the Mantel test analysis showed that there was a significant correlation between the genetic geographical structure of the wild populations (r = 0.3178, *p* < 0.05) (Figure 1). Similarly, the genetic differentiation coefficients, *G_ST_* and *N_ST_,* also indicated the existence of a genetic geographical structure. In the wild populations, the *G_ST_* was 0.843. Out of 1000 replacements, the *N_ST_* value for 950 of the replacements was 0.902. The higher value of *N_ST_* compared to the *G_ST_* value (*p* < 0.05) indicated that haplotypes with a similar genetic distance appeared in the same population or populations with a similar geographic distance. Furthermore, out of 1000 replacements, the *N_ST_* value for 990 of the replacements was 0.913. In this case, the value of *N_ST_* was also higher than that of *G_ST_* (*p* < 0.01), indicating a significant geographical genetic structure among the wild populations (Table 2). 

According to the haplotype geographical distribution map of the cultivated populations [21], there was a crossing haplotype (H3) between Songming of Kunming from Yunnan province and Huidong of Sichuan province. According to the geographical structure of the wild populations, there was a geographical isolation between the population in Central Yunnan province and that in Huidong of Sichuan province, and the haplotype of the cultivated populations appeared as a cross haplotype in these two areas. Therefore, there was no obvious geographical genetic structure. This inference was verified by the Mantel test of the cultivated populations, the results of which showed that there was no geographical genetic structure among the cultivated populations (r = 0.1629, *p* > 0.05) (Figure 1). At the same time, the *G_ST_* value of the cultivation populations was 0.754. In the 1000-replacement test, the value of *N_ST_* was 0.821 after 950 replacements (Table 2), which was higher than that of *G_ST_* (*p* > 0.05), indicating no obvious geographical genetic structure between the populations of this group. Furthermore, after 990 replacements, the value of *N_ST_* was 0.832, which was also higher than that of *G_ST_* (*p* > 0.01) (Table 2), indicating no obvious geographical genetic structure between the cultivated populations. 

**Figure 2:**
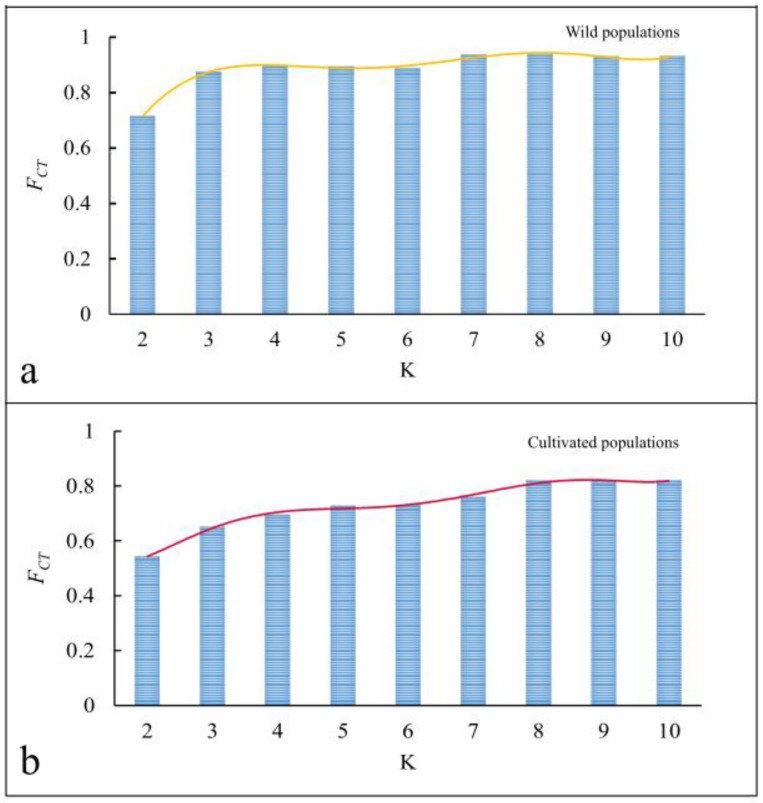
Cluster analysis diagram of the wild (**a**) and cultivated (**b**) populations based on SAMOVA analysis. Note: *F_CT_* means the intergroup genetic differentiation value.

**Figure 3:**
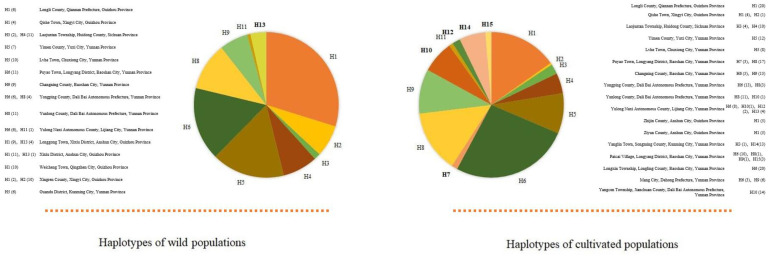
The relative proportions of different chloroplast haplotypes found in the wild and cultivated *P. polyphylla* var. *yunnanensis* populations. H1–H15: haplotype 1–haplotype 15; H in bold refers to the unique haplotypes of the wild and cultivated *P. polyphylla* var. *yunnanensis* populations. The data in parentheses indicate the number of plants.

Following the SAMOVA analysis of all wild populations and cultivated populations (Figure 2), when the K value increased from 2 to 10, the corresponding *F_CT_* value did not stabilize. A maximum *F_CT_* value indicated that there was a single population group, which meant that there was no optimal group. Following the AMOVA analysis, genetic differentiation in the wild populations and cultivated populations (76.66% and 78.86%) was observed. However, there were no genetic differences between the wild populations and cultivated populations (2.5%, *p* > 0.05) (Table 3). In terms of the relative proportions of different chloroplast haplotypes found in the wild and cultivated *P. polyphylla* var. *yunnanensis* populations, there were nine haplotypes (H1, H2, H3, H4, H5, H6, H8, H9, and H11) shared between the wild populations and the cultivated populations, one unique haplotype (H13) in the wild populations, and five unique haplotypes (H7, H10, H12, H14, and H15) in the cultivated populations (Figure 3 and Table 4).

## 4. Discussion

### 4.1. Genetic Diversity of P. polyphylla var. yunnanensis Based on cpDNA

A higher genetic diversity of plants means they can more strongly adapt to environmental changes, which is important for their survival. Genetic diversity is affected by a number of natural and human factors, such as geographical distribution, habitat destruction, and overexploitation [22]. Because chloroplast genome (cpDNA) is inherited maternally without undergoing genetic recombination, chloroplast haplotype remains almost unchanged between generations. Therefore, cpDNA is an excellent tool to study the genetic diversity of plants. In this study, there was a moderate level of cpDNA diversity (*H_T_* = 0.861), which was higher than that of *Cedrella odorata* (*H_T_* = 0.700) [23], *Juniperus przewalskii* (*H_T_* = 0.700) [24], *Alnus glutinosa* (*H_T_* = 0.773), and *Cyclobalanopsis glauca* (*H_T_* = 0.681) [25]. Petit et al. [26] determined the average cpDNA diversity of 170 plants (*H_T_* = 0.67) as measured using different molecular markers. However, *Cycas taitungensis* (*H_T_* = 0.998) [27], *Cunninghamia lanceolate* (*H_T_* = 0.952) [28], and *Scutellaria baicalensis* (*H_T_* = 0.888) all had high cpDNA diversity [29]. In this study, the moderate level of genetic diversity of wild *P. polyphylla* var. *yunnanensis* as determined by sequencing the cpDNA *trn*L–*trn*F intergenic spacing was consistent with the diversity measured using AFLP markers [18]. However, the genetic diversity of *P. polyphylla* var. *yunnanensis* was still higher than that of many other endangered plants, indicating that being endangered was not due to its own biological characteristics but rather by overexploitation or other factors.

### 4.2. Comparison of the Genetic Diversity between the Cultivated and Wild Populations

In this study, we identified 15 haplotypes in *P. polyphylla* var. *yunnanensis*, encompassing 10 haplotypes in the wild populations and 14 haplotypes in the cultivated populations (Table S1). Among them, there were nine haplotypes shared between the wild group and the cultivated group, one unique haplotype in the wild group, and five unique haplotypes in the cultivated group (Figure 3). Moreover, the genetic diversity of the cultivated populations was higher than that of the wild populations (*H_T_* = 0.900 vs. 0.861). According to the UPGMA tree of all individuals in Yunnan constructed based on AFLP markers [10], the genetic background of the cultivated *P. polyphylla* var. *yunnanensis* populations was relatively complex. Therefore, both the haplotype richness and genetic diversity of the cultivated populations were higher than those of the wild populations. However, the correlation between geographical distance and genetic distance was not strong in the cultivation populations, negating the existence of a geographical genetic structure and suggesting that the unique haplotype of the cultivation populations might have been obtained due to the mixed provenance of the cultivation populations. 

### 4.3. Selection of a Conservation Area for P. polyphylla var. yunnanensis

Wild *P. polyphylla* var. *yunnanensis* was considered an endangered species in the industrial age. Currently, *P. polyphylla* var. *yunnanensis* is classified as a national second-class endangered medicinal plant, and the development and sustainable applications of its wild resources are of far-reaching significance in order to formulate effective protection strategies. We found that *P. polyphylla* var. *yunnanensis* was widely distributed throughout Southwest China, but there were few wild individuals collected in some areas. For example, only six and four individuals were collected in Longli county (LL) and Qishe town (XY), respectively, in Xingyi city. Seven and nine individuals were collected in Yimen County and Changning County (CN), respectively, in Yunnan Province. The genetic diversity of 15 wild populations from three provinces was analyzed and compared by sequencing cpDNA fragments, the results of which showed that the wild populations in the Guizhou and Sichuan provinces had high genetic diversity; however, more studies need to be carried out in various populations.

Previously published evolution network diagrams of wild *P. polyphylla* var. *yunnanensis* showed that the H1 and H6 haplotypes are ancient haplotypes [10,21]. The genealogical analysis of wild *P. polyphylla* var. *yunnanensis* in this study indicated that Guizhou and Western Yunnan provinces might have been two independent glacial refugia, suggesting that priority should be given to these areas to protect wild *P. polyphylla* var. *yunnanensis* in these regions and strictly prohibit the excavation of land harboring wild *P. polyphylla* var. *yunnanensis* to ensure the protection of its habitat.

*P. polyphylla* var. *yunnanensis* is an important naturally growing TCM. In recent years, it has been rapidly cultivated in large quantities to meet the world’s demand for traditional Chinese medicine [30]. It takes as long as ten years for wild individuals to grow from seed germination to harvest. To protect wild resources and ensure sustainable utilization, artificial cultivation has been an effective alternative method [31]. Traditional cultivation methods entail the collection of seeds from wild populations and then planting them in similar habitats, which is an effective way to maintain the gene pool of medicinal plants [6,32]. From our survey, this traditional approach is used in only some small remote mountainous areas, while rare sites may be preserved in local distribution, such as Huidong and Sichuan (HD). Therefore, it is equally important to establish a wild *P. polyphylla* var. *yunnanensis* germplasm resource nursery for systematic relocation protection while taking local protection measures. Although we observed that these populations had high genetic diversity, hybrid provenances might produce an uneven quality of *P. polyphylla* var. *yunnanensis*. Therefore, to obtain high-quality raw materials for the development of the heavy building industry, the screening and purification of high-quality seed sources should be carried out in conjunction with relocation protection.

## 5. Conclusions

In summary, the genetic diversity and structure of 15 wild populations and 17 cultivated populations of *P. polyphylla* were analyzed by sequencing the cpDNA *trn*L-*trn*F fragments and comparing the results with those of AFLP markers. Based on the analysis of the *trn*L-*trn*F fragments, the wild populations of *P. polyphylla* had a moderate level of genetic diversity (*H_T_* = 0.861), which was consistent with the results of similar analyses using AFLP markers. A total of 15 haplotypes were identified in the 32 populations of *Paris polyphylla*; five unique haplotypes were identified in the cultivated populations, while only one unique haplotype was identified in the wild populations. Moreover, the genetic diversity of the cultivated populations was higher than that of the wild populations (*H_T_* = 0.900 vs. 0.861), which was also consistent with the results of the AFLP marker analysis. Combining the results shown in the UPGMA tree of the cultivated population grown in Yunnan province established based on AFLP markers with the results of the Mantel test of the cultivation group based on *trn*L-*trn*F fragment sequences, it was speculated that the unique haplotype of the cultivation group might have been obtained due to the mixed provenance of the cultivation populations.

## Figures and Tables

**Table 1:** Genetic diversity and genetic differentiation of *P. polyphylla* var. *yunnanensis* based on cpDNA (*trn*L-*trn*F).

Parameters	Wild Populations	Cultivated Populations	Total Populations
Haplotype	10	14	15
*H_S_*	0.135 (0.0489)	0.222 (0.0557)	0.181 (0.0376)
*H_T_*	0.861 (0.0479)	0.900 (0.0298)	0.866 (0.0269)
*G_ST_*	0.843 (0.0558)	0.754 (0.0618)	0.791 (0.0422)
*N_ST_*	0.919 (0.0450)	0.815 (0.0627)	0.860 (0.0399)

Notes: *H_S_*, haplotype diversity at the population level; *H_T_*, haplotype diversity at the species level; *G_ST_* and *N_ST_,* genetic differentiation obtained using different calculation methods. The data in parentheses indicate nucleotide diversity *π.*

**Table 2:** Genetic differentiation coefficients (*N_ST_* and *G_ST_*) for the cpDNA of *P. polyphylla* var. *yunnanensis* determined by conducting 1000 substitution U-tests.

Permutations	Wild Populations		*p*-Value	Cultivated Populations		*p*-Value
	*N_ST_*	*G_ST_*		*N_ST_*	*G_ST_*	
950	0.902	0.843	<0.05	0.821	0.754	>0.05
990	0.913	0.843	<0.01	0.832	0.754	>0.01

Notes: *G_ST_* and *N_ST_*: genetic differentiation coefficients obtained using different calculation methods.

**Table 3:** Analysis of molecular variance (AMOVA) of the 32 populations of *P. polyphylla* var. *yunnanensis* based on the cpDNA sequences.

Source of Variance	d.f.	SSD	VC	PV (%)	Fixation Indices	*p*-Value
All populations without hierarchy						
Among populations	31	122.242	0.339	76.660	*F_ST_* = 0.767	<0.001
Within populations	332	34.304	0.103	23.340		
Total	336	156.547	0.443			
Wild populations vs. cultivated populations						
Among groups	1	2.383	0.011	2.500	*F_CT_* = −0.025	>0.05
Among populations within groups	30	119.859	0.344	78.86	*F_SC_* = 0.769	<0.001
Within populations	332	34.304	0.103	20.64	*F_ST_* = 0.764	<0.001
Total	363	156.547	0.459			

Notes: *F_ST_* represents the inter-population genetic differentiation; *F_CT_* represents the intergroup genetic differentiation.

**Table 4:** Sample and geographical information of the 15 wild populations and 17 cultivated populations of *P. polyphylla* var. *yunnanensis*.

Population ID	*N*	Haplotypes	Locality	Latitude (N)/Longitude (E)	Altitude (m)	Sample No.
Wild populations						
W-LL	6	H1 (6)	Longli County, Qiannan Prefecture, Guizhou Province	26°27′47″, 106°59′33″	1080	2017CL-003
W-XY	4	H1 (4)	Qishe Town, Xingyi City, Guizhou Province	25°00′40″, 104°49′10″	1753	2017CL-041
W-HD	13	H3 (2), H4 (11)	Laojuntan Township, Huidong County, Sichuan Province	26°23′32″, 102°57′55″	2205	HY16080401
W-YM	7	H5 (7)	Yimen County, Yuxi City, Yunnan Province	24°58′38″, 102°12′52″	1893	HY16080901
W-CX	10	H5 (10)	Lvhe Town, Chuxiong City, Yunnan Province	25°07′55″, 101°22′30″	1871	HY16081401
W-LY	11	H6 (11)	Puyao Town, Longyang District, Baoshan City, Yunnan Province	25°02′09″, 099°04′108″	2298	HY16081801
W-CN	9	H9 (9)	Changning County, Baoshan City, Yunnan Province	24°94′20″, 099°56′52″	2074	HY16081901
W-YP	10	H6 (6), H8 (4)	Yongping County, Dali Bai Autonomous Prefecture, Yunnan Province	25°21′27″, 099°23′14″	1925	HY16082001
W-YL	11	H8 (11)	Yunlong County, Dali Bai Autonomous Prefecture, Yunnan Province	25°34′57″, 099°07′29″	2268	HY16082101
W-LJ	7	H6 (6), H11 (1)	Yulong Naxi Autonomous County, Lijiang City, Yunnan Province	27°01′94″, 100°22′01″	3200	HY16082201
W-LG	13	H1 (9), H13 (4)	Longgong Town, Xixiu District, Anshun City, Guizhou Province	26°05′42″, 105°52′43″	1178	HY16071301
W-XX	12	H1 (11), H13 (1)	Xixiu District, Anshun City, Guizhou Province	26°15′50″, 106°00′35″	1410	HY16071501
W-QZ	10	H1 (10)	Weicheng Town, Qingzhen City, Guizhou Province	26°44′43″, 106°22′57″	1363	HY16071601
W-XR	12	H1 (2), H2 (10)	Xingren County, Xingyi City, Guizhou Province	25°32′46″, 105°27′35″	1515	HY16072001
W-GD	6	H5 (6)	Guandu District, Kunming City, Yunnan Province	24°59′19″, 102°58′43″	2308	HY16080701
Cultivated populations						
C-LL	20	H1 (20)	Longli County, Qiannan Prefecture, Guizhou Province	26°27′47″, 106°59′33″	1080	HY16071703
C-XY	5	H1 (4), H2 (1)	Qishe Town, Xingyi City, Guizhou Province	25°00′40″, 104°49′10″	1753	HY16072103
C-HD	14	H3 (4), H4 (10)	Laojuntan Township, Huidong County, Sichuan Province	26°23′43″, 102°58′06″	2073	HY16080301
C-YM	12	H5 (12)	Yimen County, Yuxi City, Yunnan Province	24°58′38″, 102°12′52″	1893	HY16080903
C-CX	8	H5 (8)	Lvhe Town, Chuxiong City, Yunnan Province	25°07′55″, 101°22′30″	1871	HY16081403
C-LY	20	H7 (3), H8 (17)	Puyao Town, Longyang District, Baoshan City, Yunnan Province	25°02′09″, 099°04′08″	2298	HY16081601
C-CN	20	H6 (5), H9 (15)	Changning County, Baoshan City, Yunnan Province	24°94′20″, 099°56′52″	2074	HY16081903
C-YP	15	H6 (13), H8(3)	Yongping County, Dali Bai Autonomous Prefecture, Yunnan Province	25°21′08″, 099°23′06″	1829	HY16082003
C-YL	12	H8 (11), H10 (1)	Yunlong County, Dali Bai Autonomous Prefecture, Yunnan Province	25°34′57″, 099°07′29″	2172	HY16082005
C-LJ	15	H6 (8), H10(1), H12 (2), H13 (4)	Yulong Naxi Autonomous County, Lijiang City, Yunnan Province	27°01′94″, 100°22′01″	3200	HY16082203
C-ZJ	5	H1 (5)	Zhijin County, Anshun City, Guizhou Province	26°48′33″, 105°38′57″	1369	HY16071201
C-ZY	5	H1 (5)	Ziyun County, Anshun City, Guizhou Province	25°59′01″, 106°04′53″	1148	HY16071401
C-SM	14	H3 (1), H14(13)	Yanglin Town, Songming County, Kunming City, Yunnan Province	25°09′25″, 103°02′31″	1996	HY16080801
C-BC	15	H6 (10), H8(1), H9(1), H15(3)	Paicai Village, Longyang District, Baoshan City, Yunnan Province	25°11′50″, 099°19′15″	2321	HY16081603
C-LX	20	H6 (20)	Longxin Township, Longling County, Baoshan City, Yunnan Province	24°32′12″, 098°46′54″	1842	HY16081701
C-MS	9	H6 (3), H9 (6)	Mang City, Dehong Prefecture, Yunnan Province	24°29′24″, 098°20′11″	1936	HY16081703
C-JC	14	H10 (14)	Yangcen Township, Jianchuan County, Dali Bai Autonomous Prefecture, Yunnan Province	26°48′42″, 099°80′75″	2944	HY16082301

## Data Availability

All data supporting the findings of this study are included in this article.

## Supplementary Materials

The following supporting information can be downloaded at: https://www.mdpi.com/article/10.3390/genes14091754/s1, Table S1: Collecting information of 15 wild populations and 17 cultivated populations of *P. polyphylla* var. *yunnanensis.*
