# Supplementary material for: Population Genetic Analysis of Paris polyphylla var. yunnanensis Based on cpDNA Fragments
Source: Genes (Basel). 2023 Sep 2;14(9):1754. doi: 10.3390/genes14091754 (PMC10530812; doi:10.3390/genes14091754)
Supplement: Supplementary file 1 [file genes-14-01754-s001.zip › genes-2545061-supplementary.pdf]

Supplementary table

Table S1. Collecting information of 15 wild populations and 17 cultivated populations of *P. polyphylla* var. *yunnanensis*

Population ID	Locality	Latitude(N) / Longitude(E)	Altitude (m)	Sample size	Sample No
Wild populations					
W-LL	Longli County, Qiannan Prefecture, Guizhou Province	26°27′47″,106°59′33″	1080	6	2017CL-003
W-XY	Qishe Town, Xingyi City, Guizhou Province	25°00′40″,104°49′10″	1753	4	2017CL-041
W-HD	Laojuntan Township, Huidong County, Sichuan Province	26°23′32″,102°57′55″	2205	18	HY16080401
W-YM	Yimen County, Yuxi City, Yunnan Province	24°58′38″,102°12′52″	1893	7	HY16080901
W-CX	Lvhe Town, Chuxiong City, Yunnan Province	25°07′55″,101°22′30″	1871	10	HY16081401
W-LY	Puyao Town, Longyang District, Baoshan City, Yunnan Province	25°02′09″,099°04′108″	2298	11	HY16081801
W-CN	Changning County, Baoshan City, Yunnan Province	24°94′20″,099°56′52″	2074	9	HY16081901
W-YP	Yongping County, Dali Bai Autonomous Prefecture, Yunnan Province	25°21′27″,099°23′14″	1925	10	HY16082001
W-YL	Yunlong County, Dali Bai Autonomous Prefecture, Yunnan Province	25°34′57″,099°07′29″	2268	16	HY16082101
W-LJ	Yulong Naxi Autonomous County, Lijiang City, Yunnan Province	27°01′94″,100°22′01″	3200	7	HY16082201
W-LG	Longgong Town, Xixiu District, Anshun City, Guizhou Province	26°05′42″,105°52′43″	1178	13	HY16071301
W-XX	Xixiu District, Anshun City, Guizhou Province	26°15′50″,106°00′35″	1410	17	HY16071501
W-QZ	Weicheng Town, Qingzhen City, Guizhou Province	26°44′43″,106°22′57″	1363	10	HY16071601
W-XR	Xingren County, Xingyi City, Guizhou Province	25°32′46″,105°27′35″	1515	12	HY16072001
W-GD	Guandu District, Kunming City, Yunnan Province	24°59′19″,102°58′43″	2308	7	HY16080701
Cultivated populations					
C-LL	Longli County, Qiannan Prefecture, Guizhou Province	26°27′47″,106°59′33″	1080	20	HY16071703
C-XY	Qishe Town, Xingyi City, Guizhou Province	25°00′40″,104°49′10″	1753	5	HY16072103
C-HD	Laojuntan Township, Huidong County, Sichuan Province	26°23′43″,102°58′06″	2073	20	HY16080301
C-YM	Yimen County, Yuxi City, Yunnan Province	24°58′38″,102°12′52″	1893	13	HY16080903
C-CX	Lvhe Town, Chuxiong City, Yunnan Province	25°07′55″,101°22′30″	1871	14	HY16081403
C-LY	Puyao Town, Longyang District, Baoshan City, Yunnan Province	25°02′09″,099°04′08″	2298	20	HY16081601
C-CN	Changning County, Baoshan City, Yunnan Province	24°94′20″,099°56′52″	2074	20	HY16081903
C-YP	Yongping County, Dali Bai Autonomous Prefecture, Yunnan Province	25°21′08″,099°23′06″	1829	15	HY16082003
C-YL	Yunlong County, Dali Bai Autonomous Prefecture, Yunnan Province	25°34′57″,099°07′29″	2172	13	HY16082005
C-LJ	Yulong Naxi Autonomous County, Lijiang City, Yunnan Province	27°01′94″,100°22′01″	3200	16	HY16082203
C-ZJ	Zhijin County, Anshun City, Guizhou Province	26°48′33″,105°38′57″	1369	5	HY16071201
C-ZY	Ziyun County, Anshun City, Guizhou Province	25°59′01″,106°04′53″	1148	5	HY16071401
C-SM	Yanglin Town, Songming County, Kunming City, Yunnan Province	25°09′25″,103°02′31″	1996	20	HY16080801
C-BC	Paicai Village, Longyang District, Baoshan City, Yunnan Province	25°11′50″,099°19′15″	2321	15	HY16081603
C-LX	Longxin Township, Longling County, Baoshan City, Yunnan Province	24°32′12″,098°46′54″	1842	20	HY16081701
C-MS	Mang City, Dehong Prefecture, Yunnan Province	24°29′24″,098°20′11″	1936	15	HY16081703
C-JC	Yangcen Township, Jianchuan County, Dali Bai Autonomous Prefecture, Yunnan Province	26°48′42″,099°80′75″	2944	10	HY16082301
